# Transcriptome Analysis of Two Rice Varieties Contrasting for Nitrogen Use Efficiency under Chronic N Starvation Reveals Differences in Chloroplast and Starch Metabolism-Related Genes

**DOI:** 10.3390/genes9040206

**Published:** 2018-04-11

**Authors:** Subodh Kumar Sinha, Amitha Mithra Sevanthi V., Saurabh Chaudhary, Punit Tyagi, Sureshkumar Venkadesan, Manju Rani, Pranab Kumar Mandal

**Affiliations:** 1 ICAR-National Research Centre on Plant Biotechnology, New Delhi 110012, India; subsinha@gmail.com (S.K.S.); amithamithra.nrcpb@gmail.com (A.M.S.V.); saurabh.biozone@gmail.com (S.C.); punittyagi88@gmail.com (P.T.); sureshkumarv1996@gmail.com (S.V.); sarswat09@gmail.com (M.R.); 2 School of Human and Life Sciences, Canterbury Christ Church University, Canterbury CT1 1QU, UK

**Keywords:** nitrogen-responsive genes, rice, RNA-seq, nitrogen use efficiency, DEG-QTL integration

## Abstract

The nitrogen use efficiency (NUE) of crop plants is limited and enhancing it in rice, a major cereal crop, would be beneficial for farmers and the environment alike. Here we report the genome-wide transcriptome analysis of two rice genotypes, IR 64 (IR64) and Nagina 22 (N22) under optimal (N+) and chronic starvation (N−) of nitrogen (N) from 15-day-old root and shoot tissues. The two genotypes were found to be contrasting in their response to N−; IR64 root architecture and root dry weight remained almost equivalent to that under N+ conditions, while N22 showed high foraging ability but a substantial reduction in biomass under N−. Similarly, the photosynthetic pigments showed a drastic reduction in N22 under low N, while IR64 was more resilient. Nitrate reductase showed significantly low specific activity under N− in both genotypes. Glutamate synthase (GOGAT) and citrate synthase CS activity were highly reduced in N22 but not in IR64. Transcriptome analysis of these genotypes revealed nearly double the number of genes to be differentially expressed (DEGs) in roots (1016) compared to shoots (571). The response of the two genotypes to N starvation was distinctly different reflecting their morphological/biochemical response with just two and eight common DEGs in the root and shoot tissues. There were a total of 385 nitrogen-responsive DEGs (106 in shoots and 279 in roots) between the two genotypes. Fifty-two of the 89 DEGs identified as specific to N22 root tissues were also found to be differentially expressed between the two genotypes under N−. Most of these DEGs belonged to starch and chloroplast metabolism, followed by membrane and signaling proteins. Physical mapping of DEGs revealed 95 DEGs in roots and 76 in shoots to be present in quantitative trait loci (QTL) known for NUE.

## 1. Introduction

Nitrogen (N), being the constituent of most biomolecules, viz. amino acids, nucleotides, proteins, chlorophyll, and many plant hormones, it is considered the major essential nutrient required for plant growth and development [1,2]. Plants exhibit various changes in phenotype under N starvation, including reduced seed production (yield), leaf chlorosis, stunted growth, modulation in root architecture, etc., underlining the importance of N to plant growth and development [3]. In the last few decades, the development of N-responsive varieties and the extensive use of N fertilizers has resulted in increased biomass and subsequently the yield of crop plants [1,4]. However, of the total applied N fertilizer, plants are able to use only 30–40%, with the rest of the N fertilizers being lost to the atmosphere, groundwater, and rivers through various physicochemical processes, resulting in economic loss to farmers. The loss of N also results in the eutrophication of fresh water, the acidification of soil, and the release of greenhouse gases like nitrous oxide (around 300 times more toxic than CO_2_), leading to adverse impact on the environment [5,6]. The increase in the world’s population from 5 billion to 9 billion in the last 50 years demands increased production of staple foodstuffs. This in turn requires a huge quantity of N fertilizers, notwithstanding the fact that the production of N is an energy-demanding process. Thus, increased efficiency of N application in plants would not only result in higher crop yield under limited N supply, benefiting the farmers via higher net profit, but also mitigate the environmental risks arising due to an excess of fertilizers used on agricultural land. In this context, improving the nitrogen use efficiency (NUE) of rice, a dominant dietary source in almost every part of the world, would be worthwhile.

NUE of plants, in general, is defined as their efficiency at utilizing N from the soil. NUE has two major component traits, namely N uptake and N utilization. To be an N-efficient plant, both components are crucial. N uptake is mainly determined by various N transporters and probably also by the root architecture of the plant, whereas utilization is determined by assimilation, mobilization, and remobilization of the assimilated N for the purpose of economic yield. Agriculturally important crops take up N mainly in the form of nitrate (NO_3_^−^) and ammonium (NH_4_^+^) ions from well-fertilized soils. Among them, NO_3_^−^ ions act not only as a nutrient but also as signal molecules, inducing the expression of many genes including N transport and metabolizing genes, e.g., nitrate transporters (NRT1 and NRT2), nitrate reductase (NR), nitrite reductase (NiR), glutamine synthetase (GS), and glutamate synthase (GOGAT) [7,8,9]. Under a low supply of N, high-affinity transporters NRT2 and NRT3 play a significant role in N uptake, as demonstrated in maize [10].

Genome-wide expression analysis is an attractive approach to understanding complex traits like NUE. For genome-wide transcription profiling, RNA sequencing (RNA-seq), a high-throughput sequencing technology, is the best approach and has replaced microarray even in model plants like rice and *Arabidopsis*, which have high-quality whole-genome sequence information available [11,12]. In the last two decades, efforts have been made to understand the molecular and physiological basis of plants grown under N stress, which resulted in the identification of a large number of differentially expressed genes (DEGs) under limited N supply in many crop plants, including rice [13,14], soybeans [15], sorghum [16], and tea [17]. Most of these studies have concentrated on studying the global gene expression in a single genotype under low and optimal nitrogen (ammonia or nitrate), except the one on tea, where two genotypes of tea were compared for their responses under low and optimal ammonical nitrogen [13,14,15,16,17]. Global gene expression and comparative analysis of genotypes contrasting for NUE would aid in narrowing down the candidate genes. Moreover, a huge volume of literature is available on quantitative trait loci (QTL) affecting NUE in rice [18,19,20]. Integration of these two datasets (QTL and DEGs in contrasting genotypes) has the potential to identify robust candidate genes that can be directly deployed in crop improvement for NUE [21]. In the current study, an exhaustive analysis has been conducted to identify differentially expressed genes in two rice varieties, IR 64 (IR64) and Nagina 22 (N22), and compared with the available QTL data to identify the robust candidate genes for NUE in rice.

## 2. Materials and Methods

### 2.1. Plant Materials and Treatment

Two rice genotypes, viz., IR64, a mega rice variety suitable for lowland ecosystems and N22, an upland traditional and tall rice genotype, were used in all experiments. Uniform size seeds of both varieties were surface-sterilized using 0.5% HgCl_2_ for 1 min, followed by germination in de-ionized aerated water at 25 ± 1 °C in the dark. Rice seedlings of uniform length were then transferred to pots (five plants/pot) containing a mixture of perlite and vermiculite (in 1:2 ratio *v*/*v*) and grown under natural conditions for 15 days during the rice growing season. Seedlings were grown in a Yoshida medium containing 4 mM (N+) and 0.04 mM (N−) of nitrogen using NH_4_NO_3_ as the source. For each treatment, five pots were maintained.

### 2.2. Plant Phenotyping and Enzyme Assay

On the 16th day, three plants from each pot were sampled for phenotyping and enzyme analysis. Root and shoot tissues were separated and their length, fresh weight, and dry weight were measured. The dry weight of root and shoot tissues was measured after drying them at 50 °C. Chlorophyll was extracted in dimethyl sulfoxide and the content was measured according to Hiscox and Isarelstam [22]. Root system architecture (RSA) parameters such as the total root size (TRS; sum of path length of seminal and lateral roots), main root path length of seminal root (MRP), lateral root size (LRS; sum of path length of lateral roots as fraction of TRS), first-order LR number (FOLRN; emerging from seminal and roots), second-order LR number (SOLRN; emerging from first-order LRS), and lateral root density (LRD: sum of number of total lateral root as fraction of TRS) of the plants were captured using a flatbed root scanner (Epson Perfection v700 Photo-Dual lens system, Seiko Epson Corporation, Nagano, Japan) at 400 dpi.

Enzyme assay of leaf tissues for NR, glutamate dehydrogenase (GDH), GS, GOGAT, and pyruvate kinase (PK) was carried out as per Sinha et al. [23]. NiR assay was carried out according to Joy and Hageman [24] with some modifications. The enzyme extract and reaction mixture (Tris-Cl 0.5 M, pH 7.5; sodium nitrite; methyl violagen) were mixed and the reaction was initiated by adding sodium dithionate bicarbonate, which resulted in the violet color of the reaction mixture. The nitrite content was estimated colorimetrically after the disappearance of the color upon incubation and subtracting the blank value from it. The specific activity was expressed as μmoles of NO_2_^−^ reduced mg^−1^ protein min^−1^. NADP-ICDH activity was measured by monitoring the isocitrate-dependent rate of NADP+ reduction at 340 nm [25]. One unit of activity was defined as the amount of enzyme that catalyzed the production of 1 mmol NADPH min^−1^.

The citrate synthase (CS) assay was carried out by Srere [26] with some modifications, based on the reaction between 5′,5′-dithiobis 2-nitrobenzoic acid (DTNB) and Coenzyme A (CoA-SH) to form 2-nitrobenzoic acid (TNB) that shows maximum absorbance at 412 nm. The intensity of the absorbance is proportional to the CS activity. The absorbance was measured at 412 nm using the continuous spectrophotometric rate determination method. The change in absorbance at 412 nm was recorded immediately in the UV spectrophotometer for 3 min. Enzyme activity was expressed as the change in absorbance at 412 nm g^−1^ fresh weight sample min^−1^ and specific activity was expressed as the change in absorbance at 412 nm mg^−1^ protein min^−1^.

### 2.3. RNA Extraction and Sequencing 

Total RNA from two seedlings from each pot was extracted using RNeasy Plant Mini Kit (Qiagen, Qiagen India Pvt. Ltd., New Delhi, India). RNA quality assessment was performed by using a RNA 6000 Nano assay kit in Bio analyzer 2100 (Agilent, Santa Clara, CA, USA). RNA samples from across replications were pooled in equimolar concentration before library construction. Separate libraries for each treatment (N+ and N−), tissue (root and shoot) and variety (IR64 and N22) was constructed using Truseq RNA Sample prep kit (Illumina, Woodslang, Singapore) according to the manufacturer’s protocol. Thus, a total of eight libraries were constructed and each library was represented by five biological replications. The libraries were sequenced using paired end Illumina (Hiseq^TM^ 2500) sequencing technology. The raw sequence reads were submitted to NCBI short archive reads bearing accession number SRP131558.

### 2.4. RNA Sequencing Data Processing, Read Alignment, and Analysis

The paired ends reads generated were first subjected to quality check to trim ambiguity reads, adapters, and base to remove a specified number of bases at either 3′ or 5′ end of the reads. Further, raw reads were trimmed to remove low-quality reads (with Phred Score < 30 and read length < 36 bp) using Trimmomatic-0.36 [27]. We used open-source software, namely the TopHat (Bowtie for aligning the reads with the reference genome) and Cufflinks packages [28,29] for transcript assembly and differential expression analysis [29,30]. The high-quality reads were aligned to the annotated rice reference genome [31] using default parameters. Expression analysis to identify the DEGs was performed using Cuffdiff available in the Cufflinks package [29]. A rigorous comparison at FDR (False Detection Ratio) *p* value ≤ 0.05, and log^2^ fold change ≥ 2 (for upregulation), ≤ −2 (for downregulation) was performed to select DEGs. For biologically meaningful comparisons, the DEGs between N+ and N− conditions were identified for each tissue within a genotype. Thus there were a total of four comparisons for the two genotypes. Furthermore, the two genotypes were compared separately for root and shoot tissues, under both N+ and N− conditions. For identification of N-responsive transcripts between the two genotypes, the DEGs from N+ conditions were subtracted from the total DEGs identified. For functional descriptions of the DEGs identified, the Cufflink gene IDs were first converted into both the Michigan State University (MSU) and the Rice Annotation Project Database (RAP-DB) IDs and the functional descriptions available in these two databases were made use of. For further understanding of the coordination of the gene expression under nitrogen stress, Gene Ontology (GO) terms enrichment analysis was carried out [27].

### 2.5. Validation of RNA Sequencing Results Using Real Time Reverse Transcription Polymerase Chain Reaction

Primers for 14 DEGs identified by the DEG analysis pipeline and genes known to play a role in N uptake and utilization were selected. Primers were designed using Primer 3 version 4.0.0 [32]. Total RNA was isolated from root and shoot tissues of IR64 and N22 seedlings grown under optimal (control; N+) and deficient (stress; N−) N supply. RNA quality and quantity were checked by agarose gel electrophoresis and Nanodrop spectrophotometer (Thermo Scientific, Waltham, MA, USA). To construct a first-strand complementary DNA (cDNA) template, reverse transcription reaction (25 μL) was set up using 2 μg of total RNA according to the manufacturer’s protocol (cDNA synthesis kit, Invitrogen, Carlsbad, CA, USA). Real time reverse transcription polymerase chain reaction (qRT-PCR) amplifications were performed in an optical 96-well PCR plate using a One-Step Plus Real-Time PCR system (Eppendorf realplex, Hamburg, Germany). The first-strand cDNA reaction was diluted 10-fold and 2.5 μL of it was used as a template along with Power SYBRGreen Master Mix (Applied Biosystems, Warrington, UK) and 500 nM of gene specific primer in a 25 μL qPCR reaction. The qRT-PCR cycling conditions (10 min 95 °C, 40 cycles of 15 s 95 °C and 60 s 60 °C) were followed by the generation of a melting curve (obtained by heating the PCR product from 60 °C to 95 °C) to check the specificity of amplification. The amount of actin, a constitutive transcript (endogenous control), was normalized to check the fold change in the expression of the target genes. No template control (NTC) reaction was included to check whether the amplification is genuine from the cDNA sample.

### 2.6. Co-Localization of Differentially Expressed Genes Identified with Quantitative Trait Loci for Nitrogen Use Efficiency

From the literature available in the public domain, we made a comprehensive list of all the QTLs identified for NUE in rice along with their physical locations, robustness, and contribution in explaining the phenotype variance. We localized the DEGs identified in the present study to the QTL regions and the results were visualized using MapChart2.2 [33].

## 3. Results

### 3.1. Plant Phenotyping and Enzyme Assay

#### 3.1.1. Effect of N Stress on Biomass and Chlorophyll Pigment 

**Figure 1:**
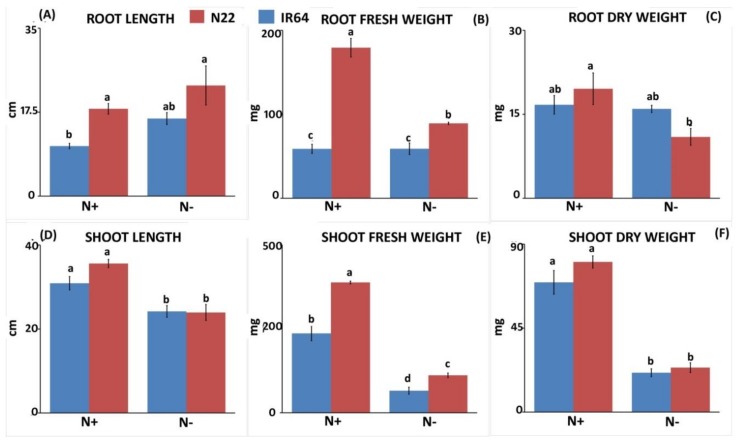
Root and shoot biomass parameters in two rice genotypes, IR 64 and Nagina 22, under optimal (N+) and low nitrogen (N) (N−). (**A**) Root length; (**B**) Root fresh weight; (**C**) Root dry weight; (**D**) Shoot length; (**E**) Shoot fresh weight; (**F**) Shoot dry weight.

**Figure 2:**
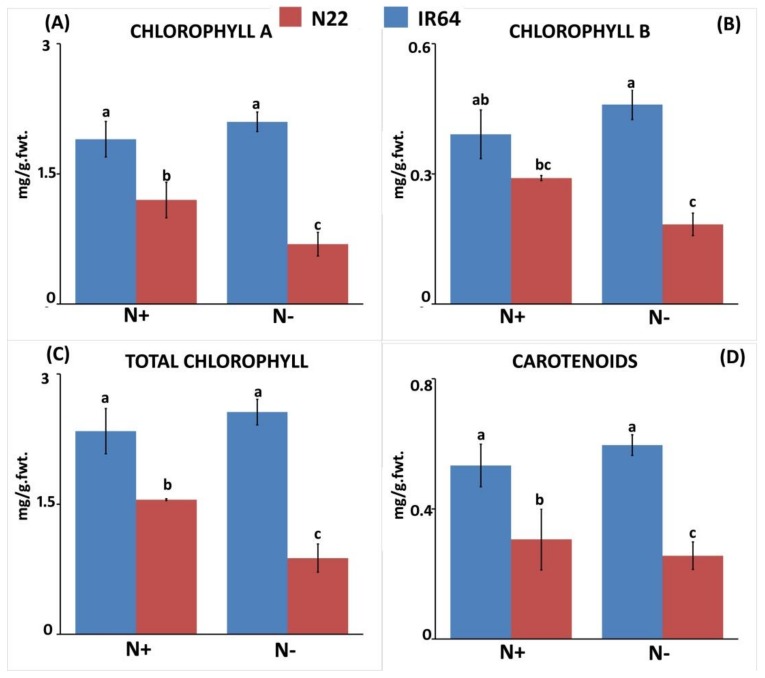
Chlorophyll and carotenoid pigments in IR 64 and Nagina 22 under optimal (N+) and low N (N−). (**A**) Chlorophyll a content; (**B**) Chlorophyll b content; (**C**) Total chlorophyll; (**D**) Carotenoid content.

N deprivation caused a substantial increase in root length in IR64, i.e., from 10.43 cm to 16.16 cm (54.94%) but not in N22, though the root length of N22 was considerably longer than IR64 under both N conditions (Figure 1A). Interestingly, the root fresh weight of IR64 was found to be less sensitive to N stress than N22, with almost negligible change (58.86 mg to 58.96 mg; 0.16%), whereas N22 showed a drastic (49.72%) reduction (Figure 1B). A similar trend was observed for root dry weight, with IR64 showing less sensitivity (4.43%) to N stress compared to N22 (43.97%). Although N22 had a higher root dry weight (19.56 mg) in N+ condition, the reduction was much greater (10.96 mg) in the N− condition compared to IR64 (16.7 mg under N+ to just 15.96 mg under N−; Figure 1C). N stress caused a significant reduction in shoot length in both genotypes; however, the reduction was more prominent in N22 (35.6 to 23.93 cm) than in IR64 (30.9 cm to 24.2 cm) (Figure 1D). Following a similar trend of shoot sensitivity to N stress, both genotypes showed an almost equal proportion of reduction for shoot fresh weight (27–28%) and shoot dry weight (29.6–30.3%; Figure 1E,F). When chlorophyll pigments (Chla, Chlb, total Chl and carotenoids) under both treatments were compared, IR64 was found not to be sensitive upon N stress compared to N22, in which all pigments were drastically reduced from N+ to N− condition except carotenoids (Figure 2). Besides being not degraded upon N stress, these pigments were present in a higher amount in IR64 than in N22 even when no stress was imposed. For instance, the Chla content was 1.9 mg g^−1^ and 1.2 mg g^−1^ under N+ while it was 2.1 mg g^−1^ and 0.69 mg g^−1^ under N− in IR64 and N22, respectively. Thus, based on both shoot and root parameters and pigment content, N22 was found to be more sensitive to N stress than IR64.

#### 3.1.2. Effect of N Stress on Root System Architecture 

**Figure 3:**
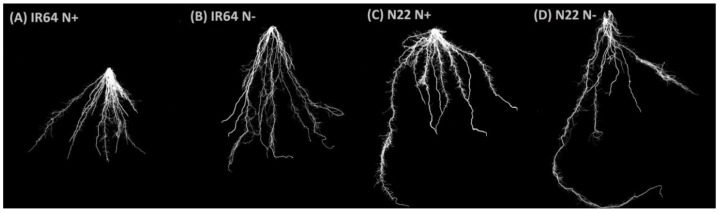

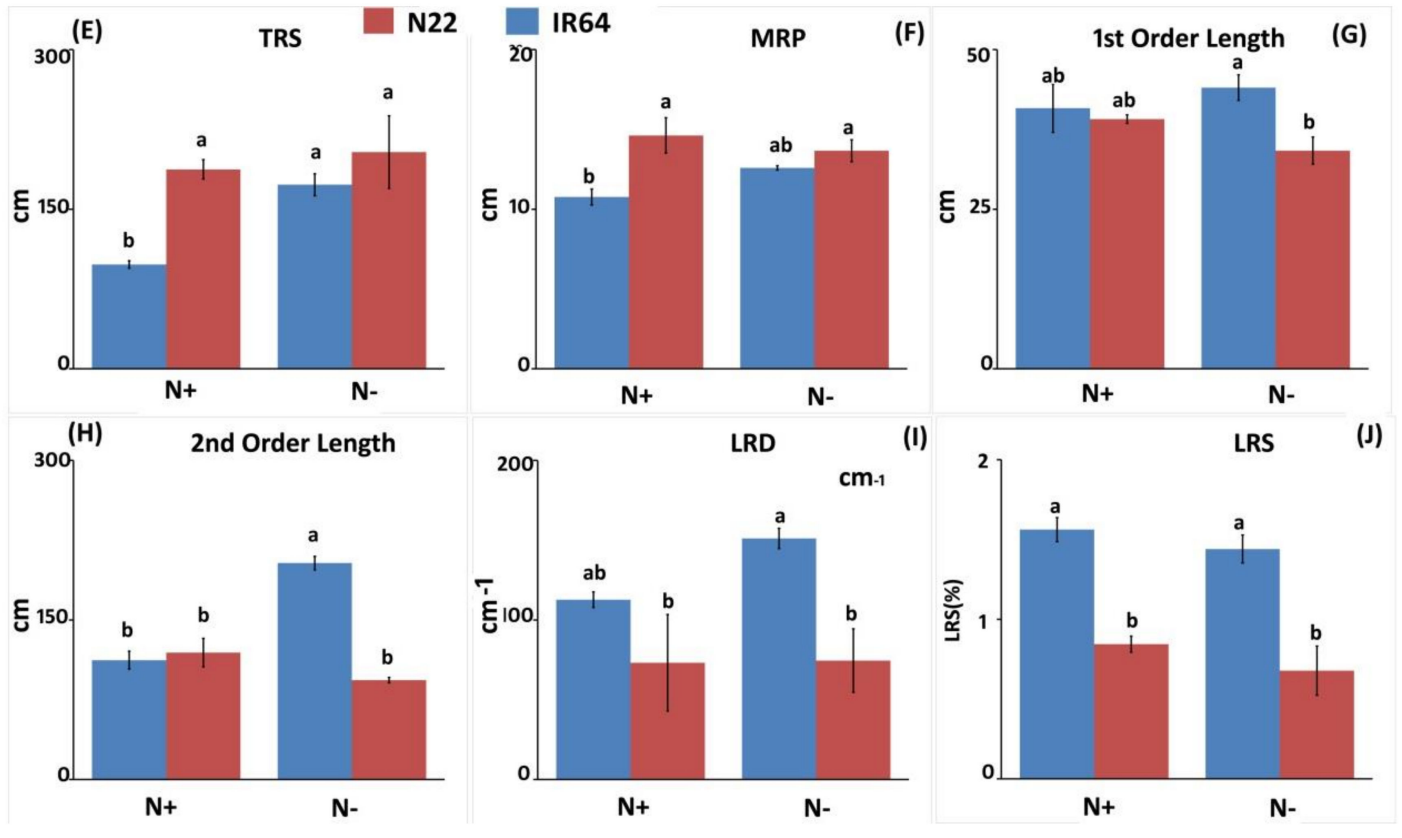
Root system architecture (RSA) parameters of the two rice genotypes, IR 64 and Nagina 22, under optimal (N+) and low N (N−). (**A**–**D**) RSA images of IR64 and N22 under N+ and N−; (**E**) Total root size; (**F**) Maximum root length; (**G**) First order root length; (**H**) Second order root length; (**I**) Lateral root density; (**J**) Lateral root size.

The root system architecture of the two genotypes under optimal and low N conditions is shown in Figure 3A–D. Although the TRS was higher in N22 under both the conditions, i.e., 187.37 cm (N+) and 203.65 cm (N−) than IR64, N stress caused a significant increase in TRS only in IR64, from 98.03 cm (N+) to 173.03 cm (N−) (Figure 3E). The MRP of IR64 slightly increased from 10.76 cm (N+) to 12.59 cm (N−), while there was a negligible change in N22 under different N conditions (Figure 3F). Both first- and second-order lateral root lengths were found to be significantly different under different N stress conditions between the two genotypes, with better FOLRN in IR64, whereas under optimal N the genotypes showed negligible differences (FOLRN 40.83 cm (N+) to 44.05 cm (N−); and SOLRN: 112.20 (Figure 3G,H). Lateral root density and size were less sensitive to N treatment; however, both these parameters were higher in IR64 in both conditions compared to N22 (Figure 3I,J). The LRD in IR64 was 112.69 (N+) and 151.11 (N−), while in N22 it was 73.17 (N+) and 74.5 (N−). LRS also showed a similar trend, with 1.56 (N+) and 1.44 (N−) in IR64 and 0.844 (N+) and 0.678 (N+) in N22.

#### 3.1.3. Effect of N Stress on Carbon and Nitrogen Metabolizing Enzymes 

**Figure 4:**
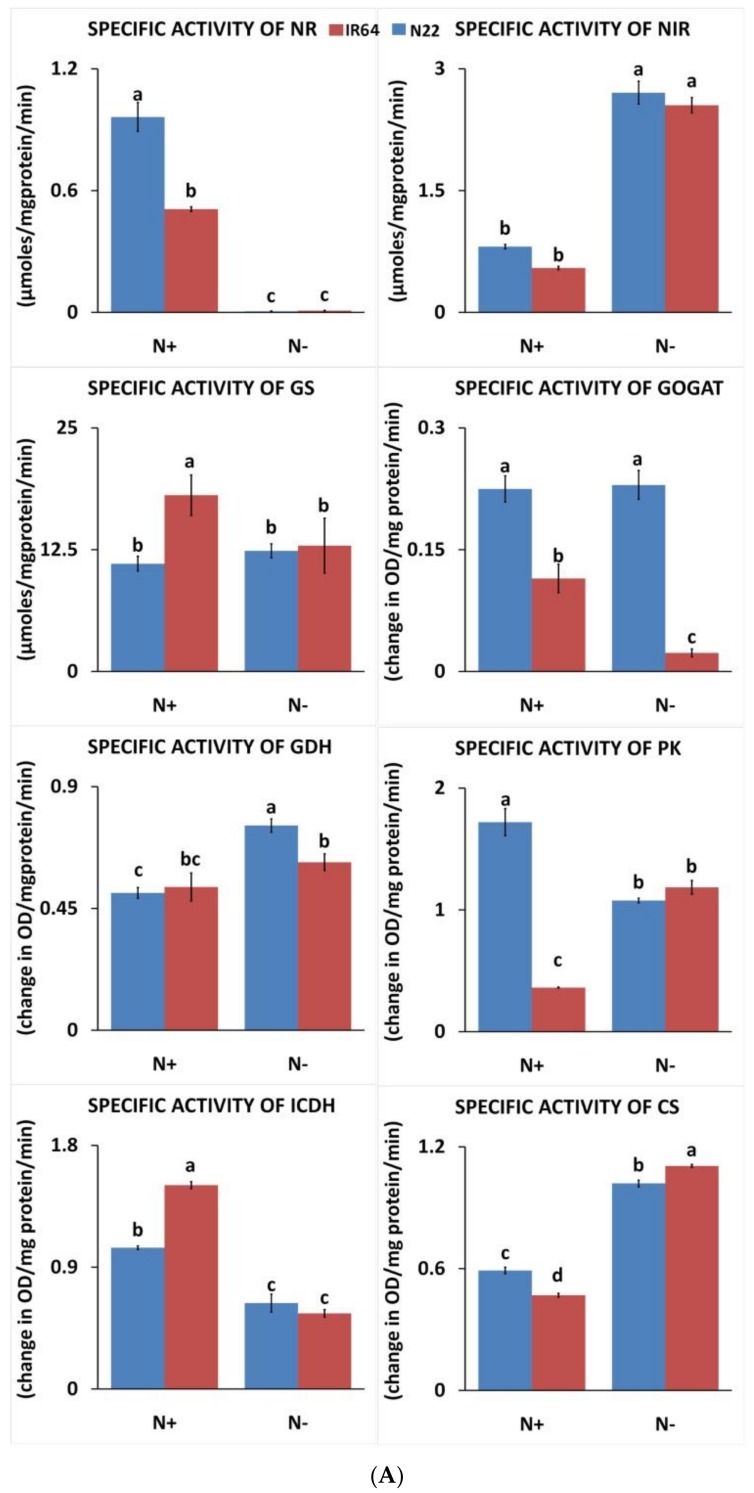

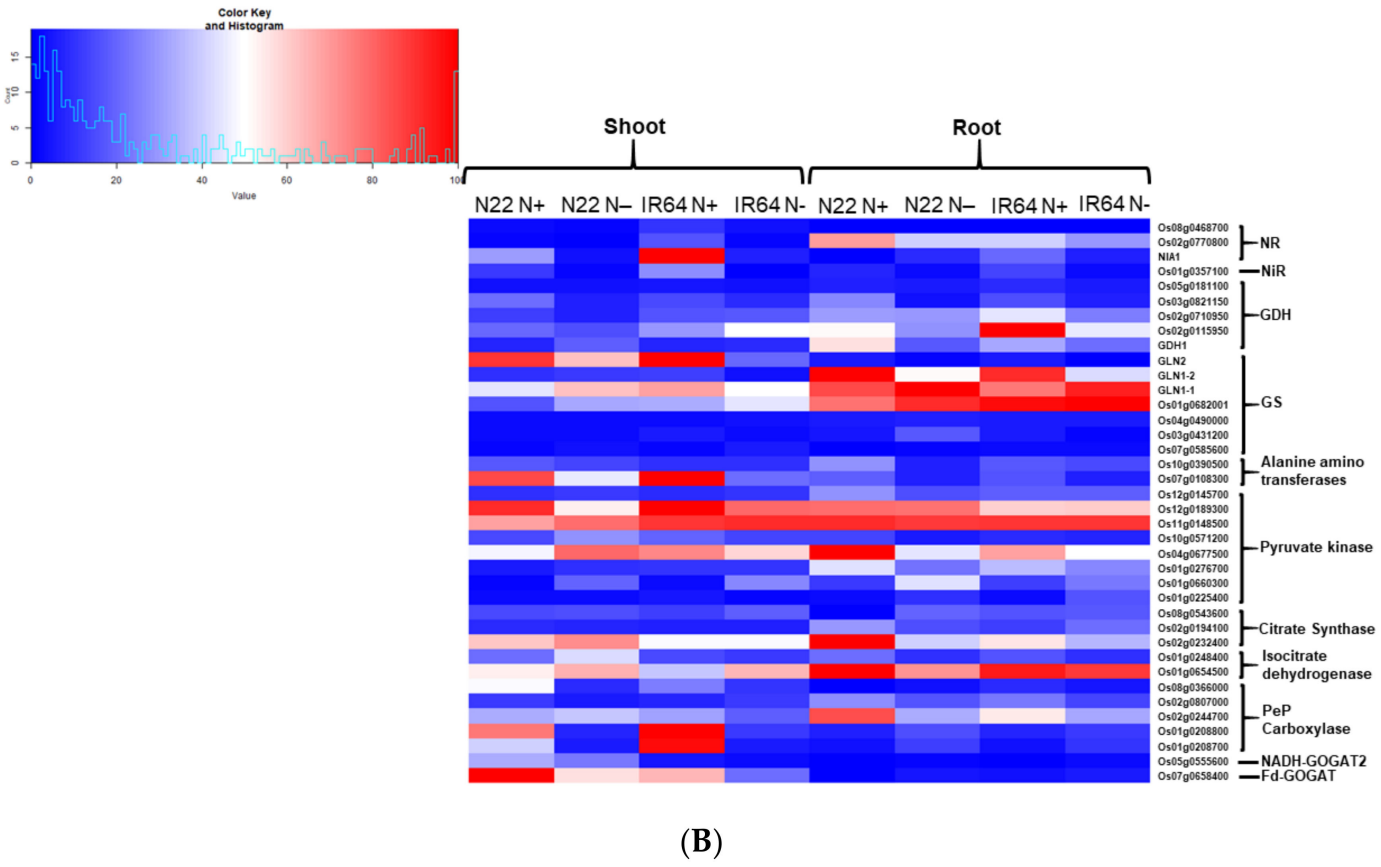
Enzyme activities and gene expression pattern in two rice genotypes, IR 64 and Nagina 22, under optimal (N+) and low N (N−). Specific enzyme activities of nitrate reductase (NR), nitrite reductase (NiR), pyruvate kinase (PK), citrate synthase (CS), glutamate synthase (GS), glutamate dehydrogenase (GDH), glutamine oxoglutarate amino transferase (GOGAT), and isocitrate dehydrogenase (ICDH) (A). The expression patterns of genes encoding for enzymes involved in nitrogen and carbon assimilation in root and shoot tissues. The heat map represents the relative expression levels of 38 genes out of the total 71 genes examined based on FPKM values (> 5 in at least one of the samples) using RNA sequencing (RNA-seq) data (B).

Of the eight enzymes studied for their specific activity, NR, PK, and ICDH showed reduced activity in both genotypes under low N while NiR and CS showed enhanced activity (Figure 4A). For GDH and GOGAT, only N22 showed reduced activity under N starvation, while IR64 had no change. On the other hand, for GDH, IR64 showed enhanced activity while N22 had no change. Thus, in cases where enzymes showed differential response between the genotypes, IR64 was the least affected or positively regulated. Interestingly, for six of the eight enzymes studied (NR, GS, GOGAT, PK, CS, and ICDH), genotype differences existed even under optimal N conditions in the study material. However, these genotype differences disappeared under N− for four enzymes (NR, GS, PK, and ICDH) but not for GOGAT and CS. NiR activity in both genotypes remained the same irrespective of the stress, though N stress resulted in significant enhancement. Among all the enzymes analyzed, NR was the most severely affected in both genotypes, with almost negligible activity (IR64: 0.0054 μmol/mg protein/min and N22: 0.0086 μmol/mg protein/min) under N stress.

### 3.2. RNA Sequencing Data Analysis: Quality Control, Assembly, and Mapping

We constructed eight libraries to compare the changes occurring at transcriptome level in the shoot and root tissues of IR64 and N22, under N starvation and optimal N conditions. Approximately 575 million raw reads were obtained from across the eight libraries, ranging from 59.3 million to maximum 85.5 million reads from each library (Table 1). After a check for read quality and removal of contamination, a huge proportion (88.56%), of high-quality (HQ) reads (510 million) remained for assembly and further downstream analysis. Thus, on average, 63.75 million HQ reads were used from each library for transcriptome analysis. The HQ reads were mapped to the rice reference genome and the detailed mapping output is summarized in Table 1. In all, 47.35% of the HQ reads uniquely mapped to the reference genome.

### 3.3. Identification of Differentially Expressed Genes and Their Validation 

A rigorous comparison at *p* value ≤ 0.05, and log^2^ fold change ≥ 2 (for upregulation), ≤ −2 (for downregulation) was made to identify the number of DEGs for different pairs of biologically meaningful comparisons. The list of DEGs, along with their fold change, annotation, and physical coordinates is presented in Table S1. The number of DEGs in root tissues (1016) was nearly twice that in shoot tissues (571) under both N treatments, suggesting the importance of roots in NUE (Table 2). In all, 209 genes in IR64 and 188 genes in N22 were identified as differentially expressed in response to N stress across both the tissues, and the number of upregulated genes was significantly higher than downregulated ones in all cases except the root tissues of IR64 (Table 2). Furthermore, the number of DEGs in roots of IR64 (between optimal and low N) was much lower (43 genes), while it was nearly three times that of shoots (116). In N22, the number of DEGs between low and optimal N was nearly identical with 89 (root) and 85 (shoot) genes. There were only two genes among the DEGs common to roots of the two genotypes in N+ and N− comparisons (LOC_Os04g56560 encoding for putative proton-dependent oligopeptide transport and LOC_Os06g44220 encoding for OsRCI2-9—putative low-temperature and salt-responsive protein) while eight such genes were in shoots (LOC_Os01g10490 encoding for keratin, type I cytoskeletal 9, LOC_Os03g53690 encoding for oxidoreductase, short-chain dehydrogenase/reductase family domain containing protein, LOC_Os04g32320 encoding for glycerophosphoryl diester phosphodiesterase family protein, LOC_Os05g44200 encoding for GDSL-like lipase/acylhydrolase, LOC_Os10g07290 encoding for glycosyl hydrolases family 17, LOC_Os11g02240 encoding for CAMK calcium/calmodulin dependent protein kinases, LOC_Os12g24020 encoding for rhodanese-like domain containing protein and LOC_Os12g41910 encoding for broad Complex BTB domain with non-phototropic hypocotyl 3 NPH3 and coiled-coil domains). Furthermore, LOC_Os01g10490 encoding for keratin, type I cytoskeletal 9 was found to be a DEG common to both root and shoot tissues.

A total of 732 DEGs were identified between the two rice genotypes under optimal N, while 855 DEGs were obtained under N stress (Table 2), signifying that the two genotype were quite different not only in their response to N stress but also in their complete genetic background. To identify the genes that were specific to N stress, the DEGs obtained under optimal N were subtracted from the DEGs from low N and this gave rise to 385 nitrogen-specific genes that were named as N-responsive genes contrasting between the two genotypes (Table 2). Of these 385 genes, 106 were specific to shoots, while 279 were specific to roots (Table 2 and Table 3). Interestingly, nearly 58.43% of the DEGs identified as specific to N22 root tissues (52 out of 89) were also found to be differentially expressed between the two genotypes under N stress (Table 3), while IR64 had only five such common genes (*Os01g0155800* encoding for glycine-rich cell wall structural protein precursor, *Os05g0163300* encoding for fasciclin domain containing protein, and three conserved hypothetical genes with no annotation: *Os06g0225600*, *Os07g0215050*, and *Os07g0560700*) out of the total of 43. This suggests that N22 roots play a major role in the unfavorable response to N stress and these 54 genes could be major candidates for improving the use efficiency of N under low N supply. In shoot tissues, no such trend could be observed, with just two (LOC_Os01g44260 encoding for dihydroflavonol-4-reductase and LOC_Os06g21270 encoding for glycine-rich protein family protein) and 12 DEGs identified in N22 and IR64, respectively, being common with those identified as DEG between the two genotypes (Table 3). Furthermore, on overall comparison of the DEGs between root and shoot tissues, we identified 25 genes, of which 12 were unknown proteins with no known function, while the rest encoded for methyl chloride transferase, acid phosphatase, ion channel containing ankyrin repeat protein, acyl-coA reductase, dihydroflavonol-4-reductase, keratin type I cytoskeletal 9 protein, HAD superfamily protein, BLE2 like protein, AP2 domain-containing protein, and GDSL-like lipase/acylhydrolase.

The genes encoding the eight enzymes studied in this report and those of alanine amino transferase and PEP carboxylase were fetched from the rice genome browser and their fragments per kilobase per million mapped reads (FPKM) values were compared and represented in a heat map (Figure 4B and Table S2). This included a set of 71 genes out of which only 38 were included in the heat map as the rest hardly had any expression under both N+ and N− conditions. Under N− conditions, NR encoding coding genes were severely downregulated, as we observed by the enzyme activity (Figure 4A,B). However, we did not observe any upregulation in *NiR* gene expression under low N, though our enzyme activity suggested otherwise. The genotype differences we observed in NADH-GOGAT even under optimal N conditions for enzyme activity were also reflected in the RNA-seq results. As enzymes like CS, GDH, and PK were encoded for by multiple genes that were also expressed in the seedling stage, the differential expression between the two genotypes under low N compared to optimal N could not be clearly deciphered. This is because the enzyme activity could be studied as a single value while the individual gene expression encoding the same protein differed. In IR64 shoots, one of the genes encoding for GDH (*Os2g115950*) showed upregulation under N− conditions but not in N22, again validating the enzyme activities measured. Similarly, upregulation was observed for two GS-encoding genes in both genotypes under low N (*GLN1-1* and *Os1g682001*). In the case of PK, though there was downregulation in both genotypes under low N, the level of downregulation was lower in IR64. The only major exception was ICDH gene expression, wherein IR64 showed no change under N− while N22 showed upregulation.

**Figure 5:**
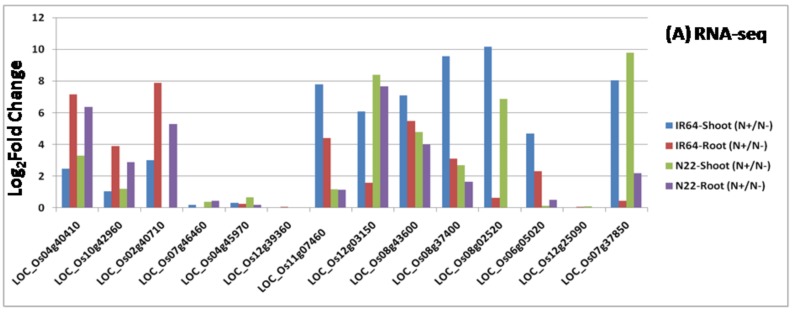

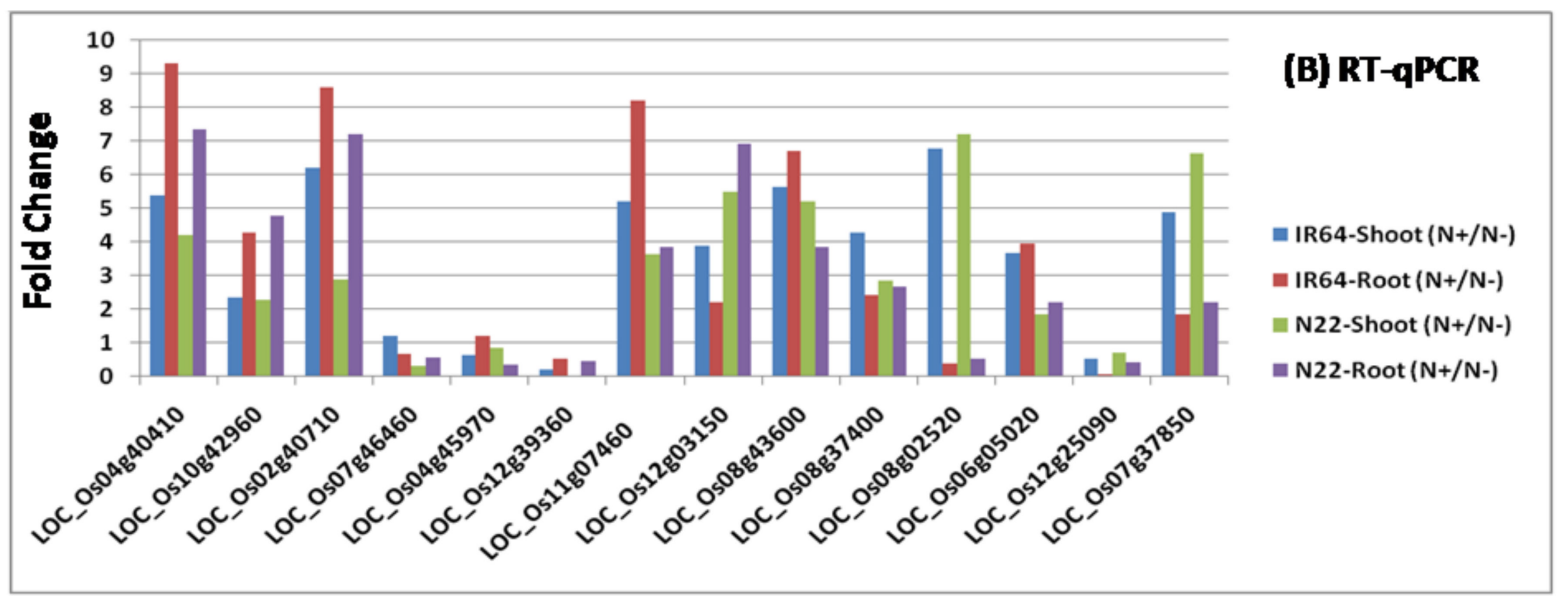
Validation of 14 differentially expressed genes (DEGs) identified from transcriptome analysis by q-PCR (**A**) RNA-seq based expression profiling; (**B**) q-PCR based expression profiling.

To validate the DEGs identified in the above analysis, 14 genes were chosen and primers for qRT-PCR analysis were synthesized (Table S3). The qRT-PCR results (Figure 5B) were in agreement with the RNA-seq data under N stress (Figure 5A) apart from a few variations.

### 3.4. Gene Ontology Enrichment Analysis for Differentially Expressed Genes

**Figure 6:**
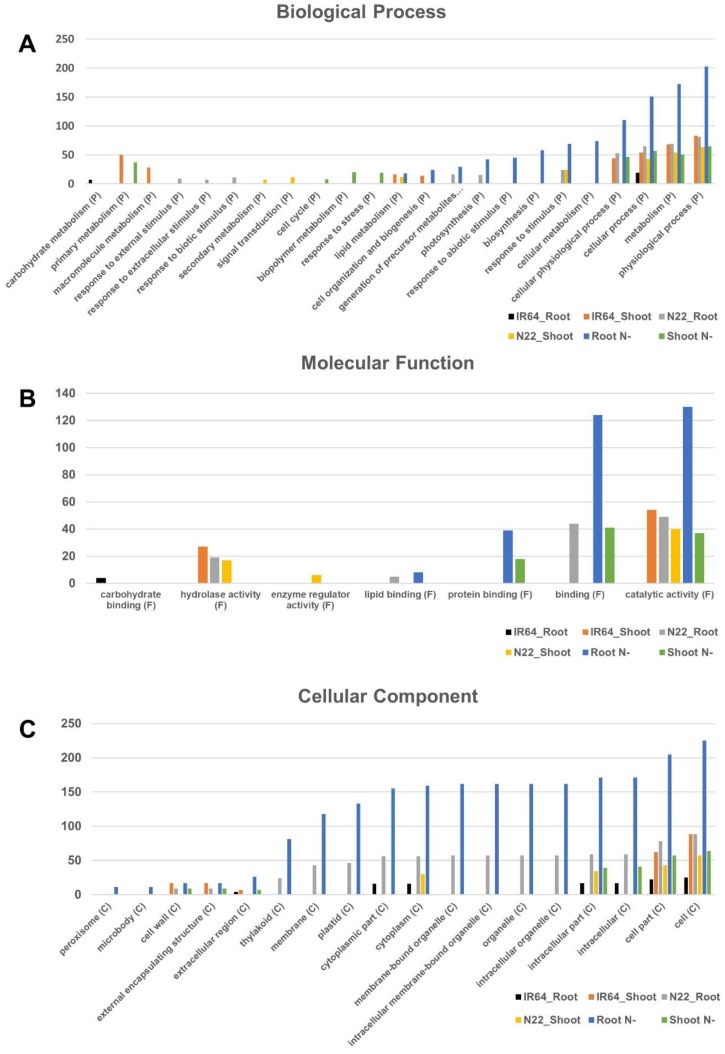
Functional classification of DEGs identified under optimal (N+) and low N (N−) in IR64, Nagina 22 and IR64 vs. N22 based on Gene Ontology (GO) terms, showing GO category distribution. (**A**) Biological process; (**B**) Molecular function; (**C**) Cellular component.

To assess whether the identified DEGs may be regulated in a functionally coordinated manner in response to N stress, the DEGs were tested for significant enrichment for a specific GO term. For this analysis, six sets of DEGs identified from root and shoot tissues of IR64, N22, and IR64 vs. N22 were used. All the DEGs identified in both genotypes (IR64 and N22) and between the genotypes (N22 N−/IR64 N−) were classified into 118 functional groups by GO analysis (Table S4). The most striking observation was the predominance of genes in root tissues across most of the functional classes of Biological Processes, Molecular Function, and Cellular Component categories (Figure 6). Under Biological Process, physiological processes, metabolism, cellular processes, and cellular physiological processes were the most abundant functional groups in most of the comparisons, with maximum abundance in the ‘between-genotype DEGs under N stress’ followed by either IR64 root or N22 shoot tissue DEGs. IR64 root tissues were conspicuous by their absence in all functional groups except for a few genes in carbohydrate metabolism and cellular processes (Figure 6A). Under the molecular function category, catalytic activity (GO:0003824) was found to be enriched in all six sets of comparisons made (Figure 6B and Table S4). Furthermore, in the root tissues of ‘between-genotype comparison’, binding (GO:0005488), both protein and lipid binding groups were enriched. IR64 and N22 roots also showed enrichment in the catalytic and binding activities. Under Cellular Component cell (GO:0005623) and cell part (GO:0044464) were found to be enriched in all six sets of comparisons. This was followed by enrichment in almost all categories, especially, intracellular, organelle, cytoplasm, membrane, and thylakoid categories in root tissues of ‘between-genotype comparison’ as in the other cases. N22 root was the next most important comparison, which was present in most of the functional groups under this category (Figure 6C). In other words, IR64 was not affected by N stress very much compared to N22.

### 3.5. Nitrogen Use Efficiency Quantitative Trait Loci in Rice and Co-Localization of Differentially Expressed Genes to the Quantitative Trait Loci Regions 

**Figure 7:**
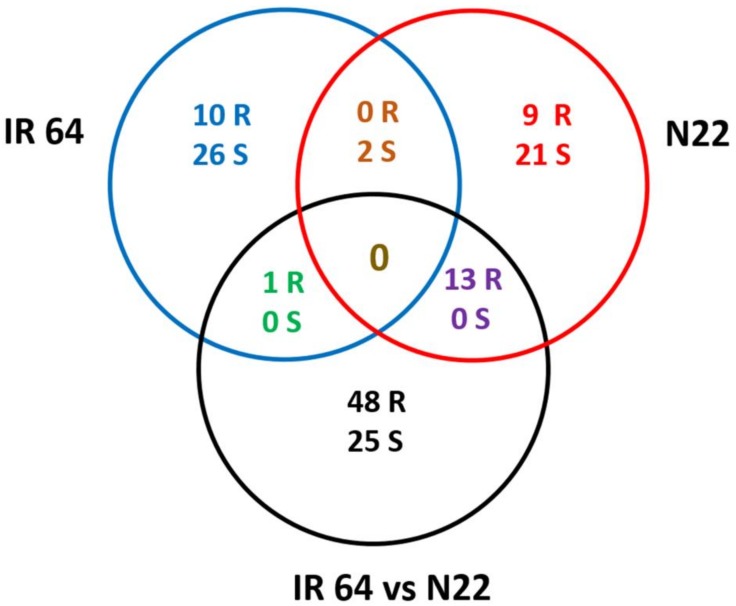
Venn diagram depicting the distribution of the DEGs identified under optimal (N+) and low N (N−) from IR64, Nagina 22 and IR64 vs. N22 co-localized with the quantitative trait loci (QTL) regions governing nitrogen use efficiency in rice.

**Figure 8:**
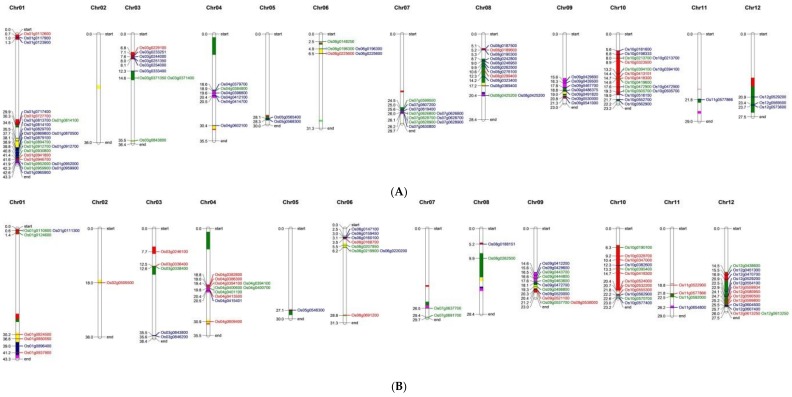
Map chart depiction of DEGs in root and shoot. DEGs identified under optimal (N+) and low N (N−) from IR64, Nagina 22 and IR64 vs. N22 and co-localized with the QTL regions governing nitrogen use efficiency in rice specific to root tissues (**A**). DEGs identified under optimal (N+) and low N (N−) from IR64, Nagina 22, and IR64 vs. N22 and co-localized with the QTL regions governing nitrogen use efficiency in rice specific to shoot tissues (**B**). Red represents genes differentially expressed in IR64 under low N, while green represents those from N22 with respective optimal N treatment as control. The genes differentially expressed between the two genotypes are shown in blue.

From the literature, the studies that identified nitrogen-responsive QTLs in rice under different N regimes were compiled (Table S5). Wherever QTLs from both conditions have been reported, only the relative trait QTLs were used for analysis [19,34,35]. In studies where such relative QTLs have not been reported, those QTLs specific to the low nitrogen regime alone were selected [18]. Those studies that attempted testing under only one nitrogen condition without emphasis on low N were removed from the analysis [36]. Furthermore, to select the robust QTLs from these reports, only those with LOD score >3 and phenotypic variance explained >10% were considered. Moreover, studies that used anonymous markers whose physical locations cannot be determined based on the respective genetic linkage maps were also not taken into account. Finally, there were 48 QTLs for relative root or shoot weight, biomass, grain yield, grain nitrogen, single plant yield, and harvest index, comprising 29 distinct QTL regions found across 12 chromosomes (Table S5). DEGs identified in the present study were co-localized on the QTL regions and there were a total of 95 such genes in roots and 76 in shoots (Figure 7 and Table S6). This was visualized separately for root and shoot tissues on Mapchart (Figure 8A,B). Of the 95 genes in identified from root tissues, 11 were specific to IR64 and 22 to N22, while the rest (62) were N-responsive genes between IR64 and N22 that could be major ones for crop improvement. Interestingly, of these 62 N-responsive genes, only one was in common with IR64 while 13 of them were in common with N22 (Figure 7). Nearly 40% of these 62 genes were uncharacterized proteins, while others were found to be related to the GOGAT cycle (malate/oxoglutarate dehydrogenase), starch biosynthesis, lipid biosynthesis, membrane proteins, etc., based on their annotations (Table S6). A similar analysis in shoot tissues revealed a nearly equal number of N-responsive genes in IR64 (28), N22 (23), and IR64 vs. N22 (25), with just two genes in common between IR64 and N22. Again, 17 of the 25 genes (68%) were expressed but uncharacterized proteins, while those with annotations showed the involvement of methyl chloride transferase, phosphate-induced protein, IQ calmodulin-binding motif family protein, and growth regulator-related protein.

**Figure 9:**
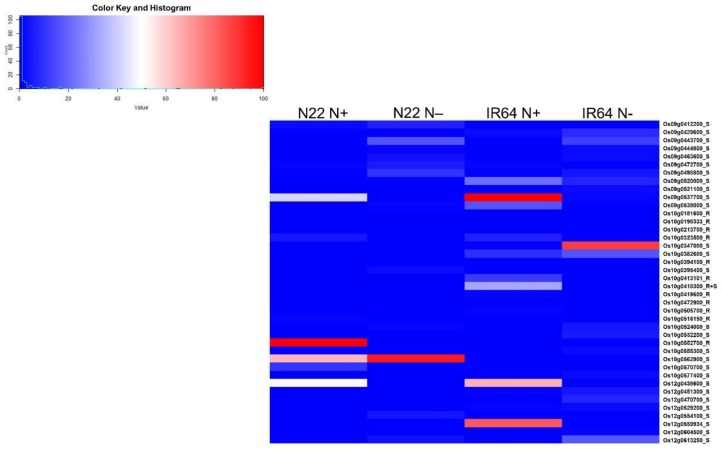
The expression patterns of the differentially expressed genes identified between two rice genotypes, Nagina 22 (N22) and IR64, from root and shoot tissues (DEGs from optimal N supply was the control) in the QTL intervals on chromosomes 9, 10, and 12. The heatmap represents the relative expression levels of 40 genes examined based on fragments per kilobase per million mapped reads (FPKM) values using RNA-seq data.

We further selected three major QTL regions on chromosomes 9, 10, and 12 and looked for their expression to see whether some could be candidate genes for NUE (Figure 9). Based on this analysis, the genes encoding for S-like ribonuclease, involved in salinity tolerance, abiotic stress response, and regulation of photomorphogenesis (*Os09g0537700*); X8 domain-containing protein (*Os09g0347000*); chlorophyllase family protein (*Os10g0419600*); and those similar to chloride channel protein (*Os12g0438600*), BTB Superoxide dismutase, copper/zinc binding gene (*Os12g0613250*), and lipoxygenase (*Os12g0559934*), with better regulation in IR64 both under optimal and low N supply than N22, could be considered as candidate genes for NUE (Figure 9 and Table S7). There were also some candidate genes that had unknown functions (*Os09g0429600*, *Os09g0498800*, *Os10g0562900*, and *Os10g0382600*). We would like to mention here that the gene expression is being compared between N22 and IR64, while the QTLs identified were from genetic backgrounds other than IR64 and N22. For more meaningful results, the QTLs for NUE between IR64 and N22 (hitherto unknown) should be considered as and when they are identified.

## 4. Discussion

Though nitrogen starvation-responsive genes have been explored in rice using medium-/high-density gene chip (microarray) and RNA-seq approaches [13,14,37], these studies confined themselves to studying the root tissues of one rice genotype at a time, which are popular high-yielding cultivars, namely Minghui 63 (indica), Dongjin, and Hejiang (japonica), and either short-term (<1 h) or medium-term (five days) responses to N starvation conditions. With huge genetic variability available in rice for all traits including response to N fertilizer application, we explored the N-responsive genes in two rice genotypes (indica and aus type), contrasting their response to chronic N starvation after confirming their response by phenotyping and enzyme studies. Unlike previous studies that sampled only root tissues for genome-wide expression analyses [13,14,37,38], except a single one where shoot tissues were studied under macronutrient (N, P, and K) deficiency [39], we sampled both root and shoot tissues in our study.

In general, N starvation affected the overall growth of both rice genotypes; however, below ground, part of IR64 was either more tolerant or was non-responsive to N stress compared to N22, i.e., IR64 kept its biomass allocation almost constant in root tissue even in N stress conditions, while N22 increased its foraging ability of nitrogen. Since in the present study a nutrient-free media (vermiculite and perlite mixture) was used for seedlings growth, which caused heterogeneous nutrient distribution, these seedlings selectively altered the root growth patterns in nutrient-rich microsites by altering root biomass allocation [40]. Interestingly, IR64 adapted to this alteration without any significant decrease in biomass parameters in N stress compared to N22.

The rice root system is mainly composed of nodal roots; however, it develops a seminal (radicle) root that emerges immediately after germination. Except for the seminal root, other RSA parameters showed a significant increase under N stress in IR64 compared to N22, wherein these root traits either showed no change or were reduced. Lateral root traits in terms of number (FOLRN, SOLRN, and LRD) were found to be modulated more profoundly than their lengths (LRS) in the case of IR64. These lateral roots play crucial roles in water and nutrient acquisition [41] by exhibiting their foraging ability in a nutrient-heterogeneous environment. Thus, IR64 perceived the N-deprivation signal more efficiently than N22, which is exhibited by modulation of its root architecture.

Chlorophyll pigments play a major role in radiation interception and hence affect leaf and canopy photosynthesis, which ultimately decides the yield of the plant. With N being a major constituent of these pigments [42], the availability of nitrogen in the leaves has a significant impact on the plant productivity. Our study demonstrated that IR64 has the unique capability to retain its total chlorophyll content even after chronic N starvation, as compared to N22. Though these pigments were estimated at a single stage, the results indicated that IR64 has a mechanism to protect their pigment system even after 15 days of N stress and hence would have better seasonal canopy apparent photosynthesis (CAP), which contributes in biomass and yield and consequently nitrogen use efficiency.

The 15-day nitrogen stress invariably caused reduced specific activity for almost all nitrogen- and carbon-metabolizing enzymes except NiR and CS (Figure 4A), which is quite obvious in the absence of corresponding substrate during each enzyme-catalyzing step. The nitrogen acquisition, transport, assimilation mobilization, etc., are highly regulated processes that follow feedback inhibition depending on the nitrogen and carbon status of the plants. NR, which catalyzes the first step of nitrate assimilation, was found to be severely reduced under nitrogen stress in both genotypes, indicating the course regulation of this enzyme under chronic N-stress. GDH was found to be increased under N stress in both genotypes, indicating its role in glutamate homeostasis in a situation of reduced GS activity [43].

To our knowledge, this is the first genome-wide expression profiling through RNA-seq report from both root and shoot tissues on chronic N starvation in rice, especially from two genotypes that are contrasting in their response to N starvation, though a similar study has recently been reported in tea [17]. Subsequently, we found a larger number of DEGs in shoot than root tissues in both genotypes under N stress, suggesting that shoot tissues are equally or more significantly affected in response to N deficiency. A comparison between genotypes also showed a substantial number of DEGs under N starvation in shoot tissues, though the number of DEGs was higher in root tissues (Table 2). Interestingly, the responses of these two genotypes were completely different to N starvation as only two genes in root tissues and eight genes in shoot tissues were found to be common between these two genotypes (Table 3). Such a difference in the array of DEGs identified in the two genotypes under low N could be due to the inherent differences in the genotypes, where one was a high-yielding genotype (IR64) and the other was a traditional tall genotype (N22). Similar differences in starch metabolism-related enzymes’ activities (AGPase- ADP glucose pyrophophorylase and starch branching enzyme II) and their transcription profiles under different N supply were reported between an N-responsive japonica genotype (cv. Nipponbare) and N-unresponsive indica genotypes, Tetep and Johna [44].

RNA-seq results supported the morphological/physiological observations: for example, chlorophyll metabolism-related genes were not differentially expressed in IR64, while 10 different genes known to function in chlorophyll metabolism, such as chlorophyllase, chlorophyll A-B binding domain-containing proteins, and many known chloroplast precursors such as lycopene epsilon cyclase, photosystem I and II related transport peptides, etc., were differentially regulated in N22. Further auxin biosynthesis was also differentially expressed in N22, unlike IR64. A comparison of differential expression between the two genotypes under N starvation also showed downregulation in these genes in addition to starch biosynthesis-related genes (Table S1, sheet 6). As 75% of the plant’s N is present in the seat of photosynthesis, i.e., chloroplasts, of which 27% are bound to Rubisco, the major enzyme of carbon assimilation, the involvement of chloroplast, the genes identified in starch, and chloroplast metabolism can be explained [45,46,47]. Furthermore, only a few genes (28 out of 279) were found to be upregulated in N22, of which 16 (57.14%) had unknown functions. Using microarray analyses of shoot tissues, similar observations wherein a few genes were upregulated and most of the differential expression occurring in basic plant development, chloroplast-related gene expression, and starch biosynthesis have been reported [39]. Most of the studies have implicated N assimilation genes, starch synthesis-related genes, and gibberellin metabolism genes in nitrogen use efficiency of plants such as rice, maize, and tea [17,44,47,48]. Interestingly, more of the LTPL lipases that are secretory proteins and signaling molecules and GDSL like lipases that are known to play a role in biotic [49] and abiotic stress tolerance such as salt tolerance [50], drought and biotic stress tolerance in transgenic plants [51] have shown differential regulation in our study. As N starvation is closely linked to starch starvation [52], a number of glycosyl hydrolases were found to be upregulated in both genotypes; however, IR64 had five different members of the glycosyl hydrolase family in shoots and one in roots highly upregulated, while N22 had just two and one members upregulated in shoot and root tissues, respectively. The N transporter genes were noticeable by their absence in any of the comparisons, probably because of the chronic N starvation of the seedlings. Still, we did observe the oxoglutarate and malate dehydrogenase and translocator genes to be N-responsive and differentially expressed in N22 but not IR64. Further studies on gene sequence comparison including promoter regions of these genes between the two genotypes could help in ascertaining their role in NUE.

Comparing the expression profiles of two contrasting genotypes for a specific trait under different treatments is supposed to help in identification of causal genes when genetic analysis (mapping) of such traits are undertaken [20,53]. Here we have identified 87 such candidate genes (62 from root and 28 from shoot) in the major QTLs regions for NUE in rice. Based on their FPKM values, some of them have also been suggested as candidate genes. We expect this will serve as a major resource for validation of the NUE-related genes in rice.

## 5. Conclusions

The current study is the first report on root and shoots transcriptome from two genotypes contrasting in their response to chronic N starvation in rice. We have demonstrated the contrasting nature of IR64 and N22 to optimal and low N supply through morphological studies including root system architecture and photosynthetic pigment estimations and the specific activity of enzymes involved in N metabolism. Furthermore, we have identified the N-responsive genes in both these genotypes and also DEGs between the two genotypes. The latter belonged to starch and chloroplast metabolism-related genes. The 95 DEGs in roots and 76 in shoots localized to the QTL intervals known for NUE in rice will serve as a resource for further detailed studies in rice and in enhancing its NUE.

## Figures and Tables

**Table 1:** Summary of trimming and read mapping results of the sequences generated from eight complementary DNA (cDNA) libraries of root (R) and shoot tissues (S) from two rice genotypes under optimal (A) and low nitrogen (D).

Library	Number of Raw Reads	Number of Trimmed Reads	Number of Uniquely Mapped Reads
IAR	66,536,324	50,191,956	37,645,788
IAS	70,824,528	55,136,806	41,531,371
IDR	71,789,678	65,991,386	12,203,558
IDS	59,320,262	54,086,464	14,888,256
NAR	85,535,858	78,152,624	40,574,926
NAS	80,808,216	74,622,520	13,694,430
NDR	68,228,978	64,247,540	40,574,926
NDS	72,947,448	67,688,471	50,455,258

I: IR 64; N: Nagina 22.

**Table 2:** Number of differentially expressed genes (DEGs) at *p* value ≤ 0.05, and log^2^ fold change: within and between genotype comparisons for shoot and root tissues under optimal (N+) and low (N−).

Comparisons	Number of Upregulated Genes	Number of Downregulated Genes	Total Number of DEGs	Roots + Shoots
IR64 root (N+/N−)	43	40	83	209
IR64 shoot (N+/N−)	102	24	126	
N22 root (N+/N−)	86	6	92	188
N22 shoot (N+/N−)	66	30	96	
IR64/N22 root (N+)	151	267	418	732
IR64/N22 shoot (N+)	218	96	314	
IR64/N22 root (N−)	294	304	598	855
IR64/N22 shoot (N−)	138	119	257	
IR64/N22 root (N-responsive)	28	251	279	385
IR64/N22 shoot (N-responsive)	73	33	106	

**Table 3:** Chromosome-wise summary of DEGs identified between optimal and low N in IR 64, Nagina 22 and the N-responsive DEGs between the two genotypes.

Chromosome	IR 64 (1)	Nagina 22 (2)	IR 64 vs. Nagina 22 (3)
Root			
1	9 (1)	12 (7)	34
2	2	15 (9)	26
3	3 (1)	6 (1)	34
4	4 (1 *)	12 (6 + 1 *)	20
5	3	4(4)	22
6	6 (1 + 1 *)	8 (5 + 1 *)	21
7	4 (2)	7 (5)	30
8	2	6 (3)	27
9	1	4 (3)	18
10	5	10 (8)	20
11	3	3 (1)	13
12	1	2 (2)	14
Total	43 (5 + 2 *)	89 (52 + 2 *)	279
Shoot			
1	10 (1 **)	10 (1 + 1 **)	9 (1 **)
2	13 (2)	10	9
3	18 (2 + 1 *)	8 (1 *)	10
4	17 (2 + 1 *)	11 (1 *)	11
5	6 (2 + 1 *)	4 (1 *)	9
6	12 (1)	6 (1)	11
7	3	6	5
8	7	5	2
9	6 (1)	9	10
10	10 (2 + 2 *)	6 (2 *)	9
11	4 (1 *)	6 (1 *)	12
12	10 (2 *)	4 (2 *)	9
Total	116 (12 + 8 * + 1 **)	85 (2 + 8 * + 1 **)	106 (1 **)
Grand total	159 (22 + 13 *)	174 (60 + 13 *)	385

Note: The number in parentheses indicates the number of DEGs common to that column and column 3. * Represents the number of DEGs common between column 1 and 2. ** Represents the number of DEGs common to all the three columns.

## Supplementary Materials

The following are available online at http://www.mdpi.com/2073-4425/9/4/206/s1, Table S1: DEGs identified between optimal and low N from IR 64 and Nagina 22 root and shoot tissues, DEGs between the two genotypes in root and shoot tissues under optimal and low N, and nitrogen-responsive genes in root and shoot tissues, Table S2: The FPKM values of the genes encoding for enzymes involved in nitrogen and carbon assimilation in root and shoot tissues of two rice genotypes, Nagina 22 (N22) and IR64 under optimal (N+) and low N (N−) supply, Table S3: Details of the primers and genes used for validation of the transcriptome results by q-PCR, Table S4: Go enrichment analysis results of the six comparisons made (DEGS from root and shoot tissues of IR64, N22 and IR64 vs. N22), Table S5: Nitrogen use efficiency QTLs known in rice and used for co-localization of N-responsive DEGs identified in the study, Table S6: Details of the DEGs co-localized with the NUE QTLs, Table S7: The FPKM values of the genes differentially expressed between the two rice genotypes, Nagina 22 (N22) and IR64 in root and shoot tissues under optimal (N+) and low N (N−) supply.
