# Supplementary material for: Transcriptome Analysis of Two Rice Varieties Contrasting for Nitrogen Use Efficiency under Chronic N Starvation Reveals Differences in Chloroplast and Starch Metabolism-Related Genes
Source: Genes (Basel). 2018 Apr 11;9(4):206. doi: 10.3390/genes9040206 (PMC5924548; doi:10.3390/genes9040206)
Supplement: Supplementary file 1 [file genes-09-00206-s001.zip › Table S3_qRT validation details.docx]

**Table S3:** Details of the primers and genes used for validation of the transcriptome results by q-PCR

| **Gene ID** | **Annotation** | **Primers (5’ to 3’)** |
| --- | --- | --- |
| LOC_Os08g02520 | OsSAUR31 - Auxin-responsive SAUR gene family member, expressed | Forward GGGCATTTCGCGGTGTA  Reverse CAGTCGAAGCCGAATTCCT |
| LOC_Os06g05020 | early nodulin 93 ENOD93 protein, putative, expressed | Forward CGCGTGAGGCGAACATT  Reverse CCATGGCAGCATCCTCAC |
| LOC_Os04g40410 | high affinity nitrate transporter, putative, expressed | Forward TGAAGGTGACCCTGTGCTA  Reverse CTGGGCGATCTTGAACTGAC |
| LOC_Os04g45970 | glutamate dehydrogenase protein, putative, expressed | Forward TCTCATCCCATGCGCTTTAG  Reverse CAGGTAATATGGTCACTCCCTTC |
| LOC_Os12g25090 | expressed protein | Forward AGGCCAAGAAGGTGATTCTC  Reverse GACGGTGTCGACGAAGATT |
| LOC_Os07g37850 | expressed protein | Forward GGCGGAGGAGAAGAAGAC  Reverse AGCCCAGGGATCAACAC |
| LOC_Os10g42960 | urea active transporter, putative, expressed | Forward CTTCCTCGCCAGCTACATC  Reverse GCCATGAGGCGATCATACA |
| LOC_Os02g40710 | ammonium transporter protein, putative, expressed | Forward CAGGGTACGACTACGACTTCT  Reverse GGAAGGCGGAGTAGATGAGATA |
| LOC_Os07g46460 | ferredoxin-dependent glutamate synthase, chloroplast precursor, putative, expressed | Forward CAAGGGAAGCCACACTACAA  Reverse CAGGACTTCTGCCACTTCTTAG |
| LOC_Os08g37400 | ZF-HD homeobox protein, putative, expressed | Forward TATCGGGAGTGCCTCAAGAA  Reverse CTCCTTGCGGTGGAAGTTAC |
| LOC_Os12g39360 | aspartic proteinase nepenthesin precursor, putative, expressed | Forward CGCTGCCGTACTACAACTC  Reverse AGATCGCAGAAGTGGATGATG |
| LOC_Os11g07460 | TCP family transcription factor, putative, expressed | Forward AGCACCACCCGTTCTACTA  Reverse CAGCAGCGGGCACTATG |
| LOC_Os12g03150 | myb-like DNA-binding domain containing protein, putative | Forward ACTGCCCATCCTCCTCATA  Reverse GTCGTAATAGCCGCTGTCATAG |
| LOC_Os08g43600 | bZIP transcription factor domain containing protein, expressed | Forward GAGCGGGAGCTGCTGTA  Reverse CGACTCGCGGTTCTTCATC |
